# Null Model and Community Structure in Multiplex Networks

**DOI:** 10.1038/s41598-018-21286-0

**Published:** 2018-02-19

**Authors:** Xuemeng Zhai, Wanlei Zhou, Gaolei Fei, Weiyi Liu, Zhoujun Xu, Chengbo Jiao, Cai Lu, Guangmin Hu

**Affiliations:** 1 School of Communication and Information Engineering, University of Electronic Science and Technology of China, Chengdu, China; 2 Faculty of Science, Engineering and Built Environment, Deakin University, 221 Burwood Highway, Burwood, VIC 3125 Australia; 3 Beijing Information Technology Institute, Beijing, China; 4 Present Address: Center for Information Geoscience, University of Electronic Science and Technology of China, Chengdu, China

## Abstract

The multiple relationships among objects in complex systems can be described well by multiplex networks, which contain rich information of the connections between objects. The null model of networks, which can be used to quantify the specific nature of a network, is a powerful tool for analysing the structural characteristics of complex systems. However, the null model for multiplex networks remains largely unexplored. In this paper, we propose a null model for multiplex networks based on the node redundancy degree, which is a natural measure for describing the multiple relationships in multiplex networks. Based on this model, we define the modularity of multiplex networks to study the community structures in multiplex networks and demonstrate our theory in practice through community detection in four real-world networks. The results show that our model can reveal the community structures in multiplex networks and indicate that our null model is a useful approach for providing new insights into the specific nature of multiplex networks, which are difficult to quantify.

## Introduction

Network science is a fundamental tool for modelling and analysing complex systems^1–3^. The general theories and approaches that have emerged from network science have provided guidelines and resulted in applications for analysis of the objects in the systems^4–6^. Therefore, research on the quantitative and qualitative features of network science has always been a focus for improving the scientific understanding of complex systems^7–10^.

Because network models capture the common features of complex systems, many network models have been proposed to study the modelling of real-world systems^11–13^. These single-network models provide a general framework of systems from different fields such as social science^14^, Internet topology^15^, bioscience^16^, engineering^17^, economics^18^, education^19^, and so on. In network science, null models are especially notable because they reveal important network properties that could not be directly quantified due to the complexity of the studied systems^20,21^. The null model concept was proposed by Maslov and Senppen^22^ and consists of a network that matches one specific graph in some of its structural features but that is otherwise taken to be a random network instance. The null model is used in comparisons to quantify complex network properties such as community structure^23,24^, assortativity^25,26^, degree correlation^27^, epidemic spreading rate^28^, motif identification^29,30^, routing efficiency^31^, pattern detection^32^, microbial diversification^33^, etc.—all of which have been shown to be significant in various complex networks. Therefore, the null model of single networks has been a powerful tool over the past few decades in analysing the nature of modelling, structures and dynamics of complex networks^34–36^.

However, the limitations of single networks have become increasingly evident over the past few years since the mass emergence of complex systems with multiple interaction layers, which are almost impossible to represent using isolated networks. Multiple relationships among objects give rise to multiplex networks in real-world systems that consist of multiple layers^37–39^. In such networks, all the relationship types are constrained by the same objects and are therefore not completely independent. Thus, each type of relationship among nodes can be described in each layer of the multiplex networks, and each network layer contains the same set of nodes. Examples of such multiplex systems include social networks involving multiple relationships from different social platforms such as Twitter, YouTube and Facebook^40^, epidemic networks with multiple diseases^41^, and Internet topologies with multiple levels from the route level to the AS level^42^. Therefore, multiplex networks, including multilayer networks^43,44^, multiscale networks^45,46^, and time-dependent networks^47–49^, are a general framework for modelling and analysing the new phenomena emerging from these multi-layered systems. The research on multiplex networks, including community detection^50^, link prediction^51^, epidemic spreading^41^, controllability^52^, synchronization^53^, and network evolution^54^, has illustrated that obvious differences exist between multiplex networks and isolated networks. For example, the synchronization state of the entire system is influenced by each layer; thus, a global unstable state may be caused by the interactions among various stable layers^55^. It is not possible to build the null model of single network for each layer of network separately because each layer is interrelated. However, the null model of multiplex networks remains unexplored, as there are few effective stochastic models that can be used to quantify the specific nature of multiplex networks.

In multiplex networks, the rich node connection information leads to redundancies in the networks, meaning that edges between the same pair of nodes could appear repeatedly in different network layers^56^. Nodes with many repeated edges are more likely to belong to the same community. For example, close friends may contact each other using different social networks such as WeChat, Twitter, and Facebook; intuitively such nodes potentially belonging to the same community. Without redundancy, the connection tightness between objects in multiplex networks could not be represented effectively and accurately. Moreover, edge redundancy leads to node redundancy in multiplex networks. The node degree of a single network cannot be used in a multiplex network due to this redundancy. Therefore, a new measure is needed to replace the node degree in multiplex networks to constrain the null model of multiplex networks.

In this paper, we propose a new general measure of nodes to fill this gap and generate a novel Null Model with Redundancy (NMR) for multiplex networks. Our goal is to describe the redundant connection relationships among nodes and provide a general framework to quantify the specific nature of multiplex networks. To achieve this, two measures, the Node Redundancy Degree (NRD) and Edge RedundanCy (ERC), are calculated based on the redundancies in multiplex networks. We build the NMR with the same NRD that exists in the original multiplex network through a configuration method. The NMR can also be explained using the traditional random-walk method. The final result is a model with an explicit edge probability under Laplacian dynamics that provides new insight into the specific nature of multiplex networks, which are difficult to quantify. Our model requires no preconditions on the systems and applies to both directed and undirected systems. We demonstrate the performance of our model by building the modularity^57^ of multiplex networks to study the community structure. The experimental results show that the community structure of multiplex networks can effectively be exposed through the NMR. Our findings fill the gap in the field of null modelling of multiplex networks and provide a powerful tool for modelling and analysing complex systems with multiple relationships in many general scientific fields.

## Results

### The Basic Model and Redundancy

In this paper, we choose an adjacency matrix to represent a network because it contains all the connection relationships in the network. A multiplex network consists of a set of networks. Therefore, we use the set of adjacency matrixes representing each isolated network in a multiplex network to preserve the complete connection information of the multiplex network. That is, *MN* = {*A*_1_, *A*_2_, …, *A*_*k*_, …, *A*_*M*_}, *k* ≤ *M*, where *M* denotes the number of networks in the multiplex network and *A*_*k*_ = (*a*_*ij*_)_*N* × *N*_ represents the adjacency matrix of each single network *k*. *N* represents the number of nodes in the network.

**Figure 1:**
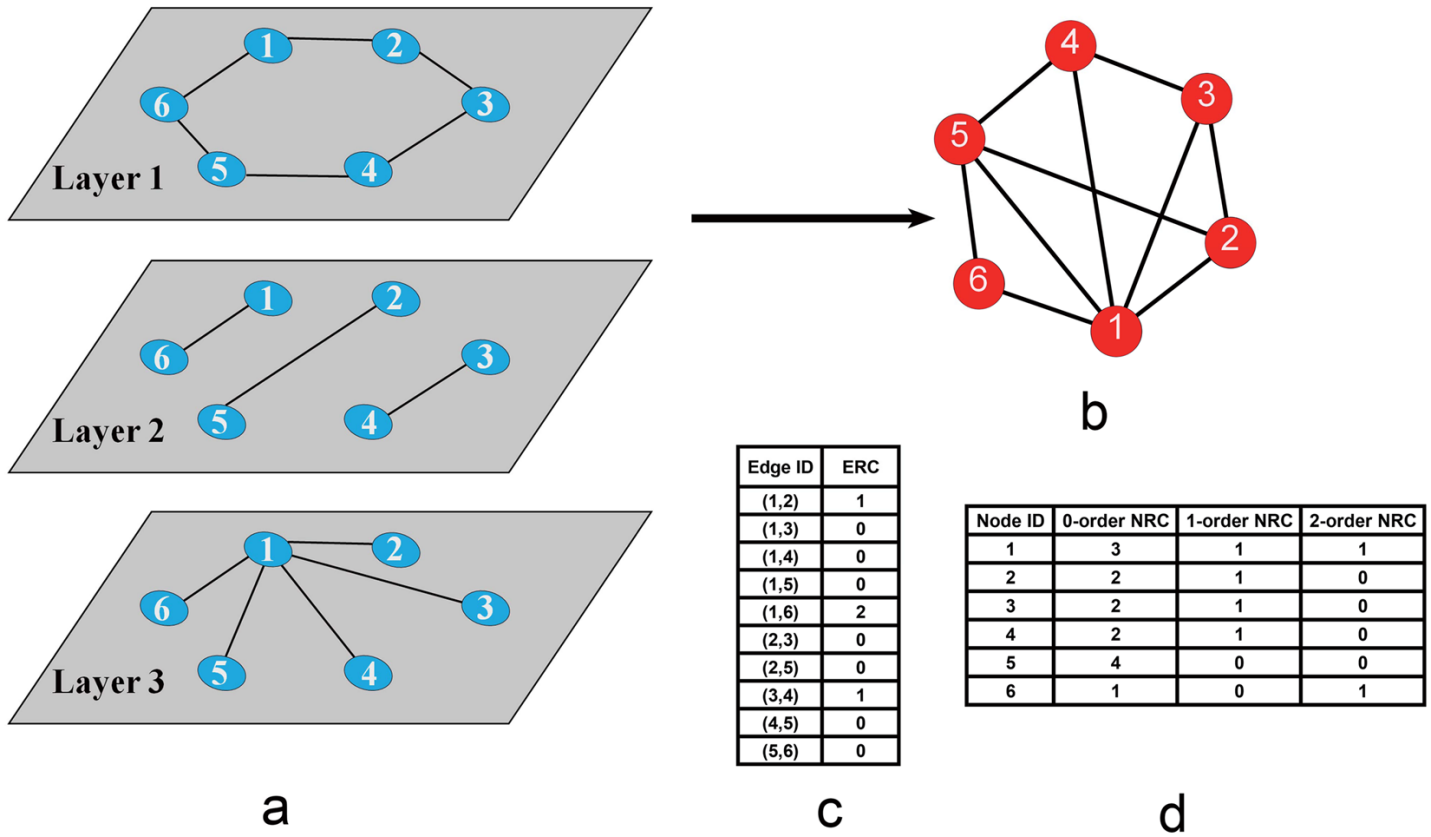
The ERC and NRD of the multiplex networks. (**a**) Three layers of the multiplex networks. (**b**) The synthetic network of the multiplex networks in (a). We combine these three networks into one network by adding an edge between two nodes if there is any edge between them in one of the three networks. (**c**) The ERC of the multiplex networks in (a). Edge (1,6) appears three times with the repeated number of two in the multiplex networks. Therefore, the ERC of edge (1, 6) is 2. (**d**) The NRD of the multiplex networks in (a). Node 1 has a 2-order NRD that is equal to 1 because there is one edge (1, 6) whose ERC is equal to 2 that is connected with node 1.

Because more than one network exists in a multiplex network, an edge between node *i* and node *j* could exist in duplicate. This redundancy represents the degree of repetition of a graph structure; therefore, this measure captures the phenomenon that a set of nodes constituting a community in one network tend to also constitute a community in other networks. Such redundancy is a basic attribute of multiplex networks. Here, we first define ERC (Fig. 1a–c) as follows:

#### Definition 1.

Edge Redundancy (ERC): The ERC refers to the number of duplicates of an edge in a multiplex network. We use *m*_*ij*_ to represent the measure

1 $${m}_{ij}=|\{k|\exists {e}_{ij}\in {E}_{k}\}|-1,$$

where *E*_*k*_ is the set of edges in layer *k* and *e*_*ij*_ is the edge between node *i* and node *j*. The formula (1) means the number of layers where there are edges between node *i* and node *j* and minus one. To a certain extent, the ERC captures the phenomenon that an edge that exists in one network tends to appear in other networks. Intuitively, the edges with high ERC values should be segmented into a community instead of between communities. Naturally, we could divide the edges into *M* groups according to the ERC.

We also define the NRD as follows:

#### Definition 2.

Node Redundancy Degree (NRD): The NRD of node *i* refers to the number of connected nodes *j* for which the ERC values of edge *m*_*ij*_ differ. We use ${r}_{i}^{m}$ to represent the *m*-order NRD of node *i*, which denotes the number of connected nodes *j* for which the ERC of edge *m*_*ij*_ is equal to *m*:

2 $${r}_{i}^{m}=|\{j|\exists {m}_{ij}=m\,\}|,\,0\le m < M$$

where *M* denotes the number of single networks in the multiplex network. The NRD ${r}_{i}^{m}$ represents the degree of the connected edge for which the ERC equals *m* (Fig. 1a,b,c). When the multiplex network degenerates to a single network, the NRD ${r}_{i}^{m}$ becomes the degree *k*_*i*_ of node *i*. Therefore, the NRD is a new parameter that measures the degrees of nodes in multiplex networks.

#### Definition 3.

Redundant Relation Matrix (RM): The RM is a matrix that describes the redundant connections between two nodes:

3 $$RM=\sum _{k}{A}_{k},\,k\le M$$

where *A*_*k*_ = (*a*_*ij*_)_*N*_ _×_ _*N*_ represents the adjacency matrix of each single network *k*. The element *rm*_*ij*_ in the matrix refers to the number of occurrences of the edge between node *i* and node *j*. That is, *rm*_*ij*_ = *m*_*ij*_ + 1. Using the *RM*, we can simplify the calculation of the ERC and the NRD as follows:

4 $${m}_{ij}=r{m}_{ij}-1$$

5 $${r}_{i}^{m}=|\{j|\exists r{m}_{ij}=m+1\}|.$$

### Null Model with Redundancy for Multiplex Networks

One of the null models of a single network proposed by Newman used the node degree *k*_*i*_ to determine the structures of random networks; later, Mahadevan proposed their higher-order representations (see Supplementary Note 1). Because NRD is an evolution of the concept of node degree for multiplex networks, we use the NRD to define null models and their higher-order representations in multiplex networks. The null model with redundancy for multiplex networks is based on the configuration model of a single network^58,59^ and dKGRAPHS^27^. The model can also be explained by Laplacian Dynamics^60^ and random walk^35^. In this model, the edge probability of the configuration method and the random walk method are unified. Based on the null model of a single network, we introduce our null model with redundancy for multiplex networks (NMR):

#### Definition 4.

Null Model with Redundancy for Multiplex Networks (NMR): The NMR is a network model that matches the original multiplex network in NRD but is otherwise taken to be a random network instance.

#### Definition 5.

*K*-Order NMR: This network model matches the original multiplex network in size and *d*-order NRD distribution *P(r)* but is otherwise taken to be an instance of a random network.

**Figure 2:**
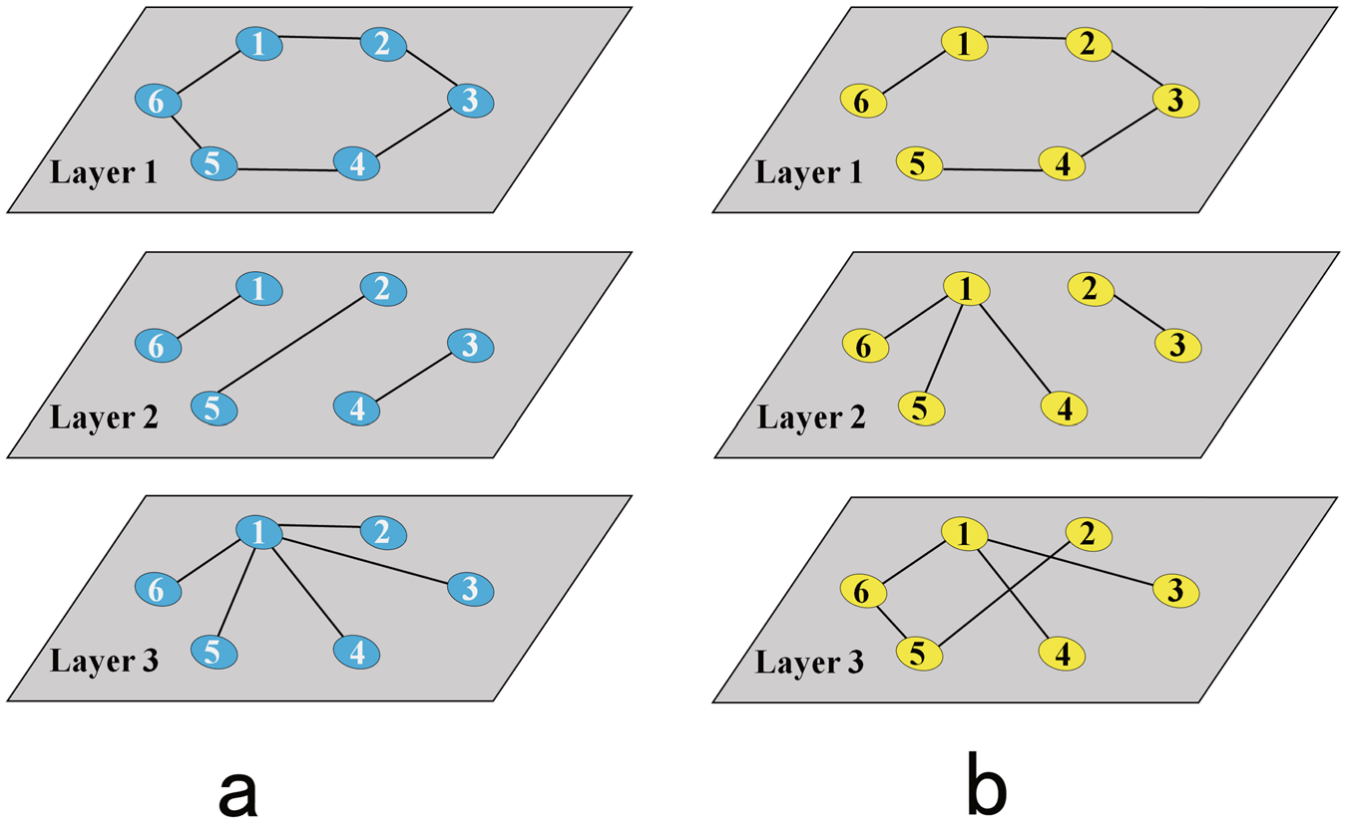
A 1-order Null Model with Redundancy of Multiplex Networks. (**a**) Three layers of the multiplex networks. (**b**) The 1-order Null Model with Redundancy of the multiplex networks in (**a**). All nodes in the model have the same 1-order NRD distribution as the original multiplex networks but the connections are random. Nodes in each layer of networks are also connected randomly under the constraints of 1-order NRD distribution. This causes the different connection between the model and the original multiplex networks.

The 1-order NMR is shown in Fig. 2. A 1-order NMR is a random model for the whole multiplex network rather than for each layer. Therefore, the aggregated information can be encoded into the multiplex structure. However, the NMR is not only a randomized aggregate version of the original network but also of each layer of the network under the constraint of the NRD. In Fig. 2, each network layer is connected differently between the NMR and the original network. The connections in each layer are also randomized—but they are not completely random. The NRD is a measure that applies to the whole multiplex network. It describes the relationships among each layer and ensures that they are not completely independent in the multiplex network. Therefore, randomization under the constraint of the NRD can randomize both the aggregated information and the information in each layer while preserving the basic relationships among each layer in a multiplex network.

Note that in multiplex networks, we use the NRD distribution instead of the degree distribution and “of the same size” means that the model has the same number of nodes *N* and number of networks *M* as the original multiplex network. Here, we provide the details of *K*-Order NMR, and a summary is shown in Table 1.

**Table 1:** The summary of k-order null model with redundancy of multiplex networks.

Tag *k-*order	Property symbol	*k-*order -distribution
*0*-order	*P*_*0*_	average NRD
1-order	*P*_1_	*P*(*r*)
2-order	*P*_2_	*P*(*r*_1_, *r*_2_)
3-order	*P*	*P*(*r*_1_, *r*_2_, *r*_3_)
*…*	…	…
*n*-order	*Pn*	*P*(*r*_1_, *r*_2_, …, *r*_*n*_)

0-Order: A random network with the same number of nodes *N*, number of networks *M*, and average NRD as in the original multiplex network.

1-Order: A random network with the same number of nodes *N*, number of networks *M*, and NRD distribution *P*_1_(*r*) as in the original multiplex network.

2-Order: A random network with the same number of nodes *N*, number of networks *M*, and 2-order NRD distribution *P*_2_(*r*_1_, *r*_2_) as in the original multiplex network.

*N*-Order: A random network with the same number of nodes *N*, number of networks *M*, and *n*-order NRD distribution *P*_*n*_(*r*_1_, *r*_2_…, *r*_*n*_) as in the original multiplex network.

### Modularity of Multiplex Networks

In this study, with the NMR, we propose the modularity of a multiplex network. Based on the modularity of a single network (see Supplementary Note 5), the modularity of a multiplex network refers to the actual number of edges within communities minus the expected number of such edges in the 1-order NMR.

In a multiplex network, the actual number of edges between node *i* and node *j* is *rm*_*ij*_ in the RM, and the expected number of such edges in the first-order NMR is

6 $${P}_{ij}=2\mu \times p(i,j)=2\mu M\sum _{m}\frac{{C}_{m+1}^{1}}{{C}_{M}^{m+1}}\frac{{(m+1)}^{2}{r}_{i}^{m}{r}_{j}^{m}}{{(2{\mu }^{m})}^{2}},$$

where *μ* is the total number of edges, *p*(*i*, *j*) is the probability of there being an edge between node *i* and node *j* in the NMR (See Methods), *M* is the number of layers in the original multiplex networks and *μ*^*m*^ is the number of *m*-ERC edges, meaning that there are *μ*^*m*^ edges whose ERC equals *m*. According to this definition, we can obtain the modularity function of a multiplex network:

7 $$\begin{array}{rcl}{Q}_{M} & = & \frac{1}{2\mu }\sum _{ij}[r{m}_{ij}-{P}_{ij}]\delta ({g}_{i},{g}_{j})\\  & = & \,\frac{1}{2\mu }\sum _{ij}[r{m}_{ij}-2\,\mu M\sum _{m}\frac{{C}_{m+1}^{1}}{{C}_{M}^{m+1}}\frac{{(m+1)}^{2}{r}_{i}^{m}{r}_{j}^{m}}{{(2{\mu }^{m})}^{2}}]\delta ({g}_{i},{g}_{j})\end{array}$$

where *g*_*i*_ refers to the community that node *i* belonging to, *δ*(*g*_*i*_, *g*_*j*_) = 1 if *g*_*i*_ = *g*_*j*_, and *δ*(*g*_*i*_, *g*_*j*_) = 0, otherwise. When the number of networks *M* is 1, the multiplex network degenerates into a single network and the modularity function of the multiplex network automatically becomes the single network modularity proposed by Newman. Thus, we can consider the modularity function of a multiplex network as an extension of single network modularity to multiple networks. Compared with the modularity of a multi-slice network, this function focuses on the impact of NRD instead of on virtual connections, which do not exist in reality. Thus, our framework is more in line with the actual structures of multiplex networks and is a more acceptable measure for analysing multiplex networks.

### Community Detection in Multiplex Networks

We first give a definition of community in a multiplex network:

#### Definition 6.

Community in a Multiplex Network: In a multiplex network, a community consists of a group of nodes that are tightly connected. Here, the tight connection means that many more edges exist within the community than among the communities. Note that each layer of a multiplex network contains the same nodes but the edges are different; the number of edges between two nodes should be calculated from all layers of the network.

**Figure 3:**
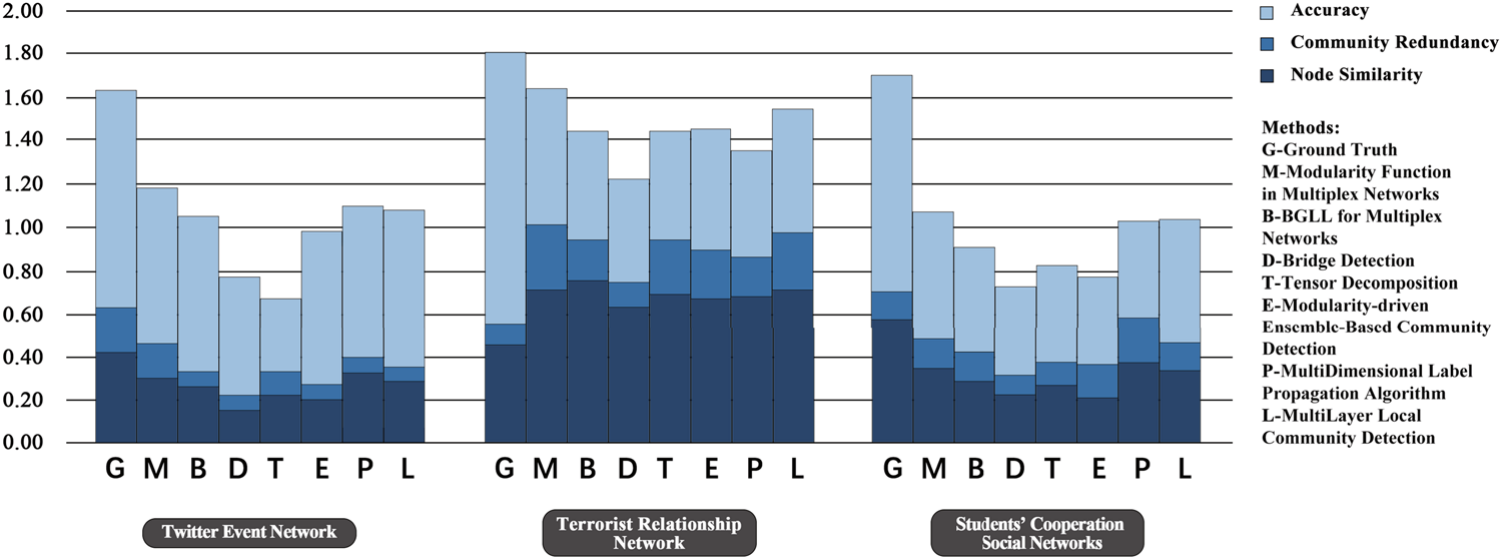
Experimental results of community detection based on real multiplex networks. The seven algorithms are modularity function in multiplex networks (MFMN), BGLL for multiplex networks after network projection (BGLLMN), bridge detection (BD), tensor decomposition for multiplex networks after network projection (TD), Modularity-driven Ensemble-Based Community Detection (M-EMCD), Multidimensional Label Propagation Algorithm (MLPA) and Multilayer Local Community Detection (ML-LCD). The three measures are node similarity, community redundancy and accuracy. The datasets are Twitter event networks, Noordin terrorist relationship networks and students’ cooperation social networks.

We executed some community-detection algorithms across the Twitter event networks, Noordin terrorist relationship networks, student-cooperation social networks and global terrorism networks. These algorithms are BGLL for multiplex networks (BGLLMN)^61,62^, bridge detection (BD)^63^,tensor decomposition for multiplex networks (TD)^64^, Modularity-driven Ensemble-Based Community Detection (M-EMCD)^65^, Multidimensional Label Propagation Algorithm (MDLPA)^66^, Multilayer Local Community Detection (ML-LCD)^67^ and our modularity function for multiplex networks (see Supplementary Note 2). Figure 3 shows the results of this quantitative comparison (see Supplementary Note 3) on three of the tested networks and indicates that the modularity function for multiplex networks results in higher-quality communities than do the other tested methods (see Supplementary Note 3). In addition, the results in Fig. 3 show that communities in real networks always have much higher redundancy, which verifies the importance of checking the redundancy in multiplex networks.

#### Twitter Event Networks

**Figure 4:**
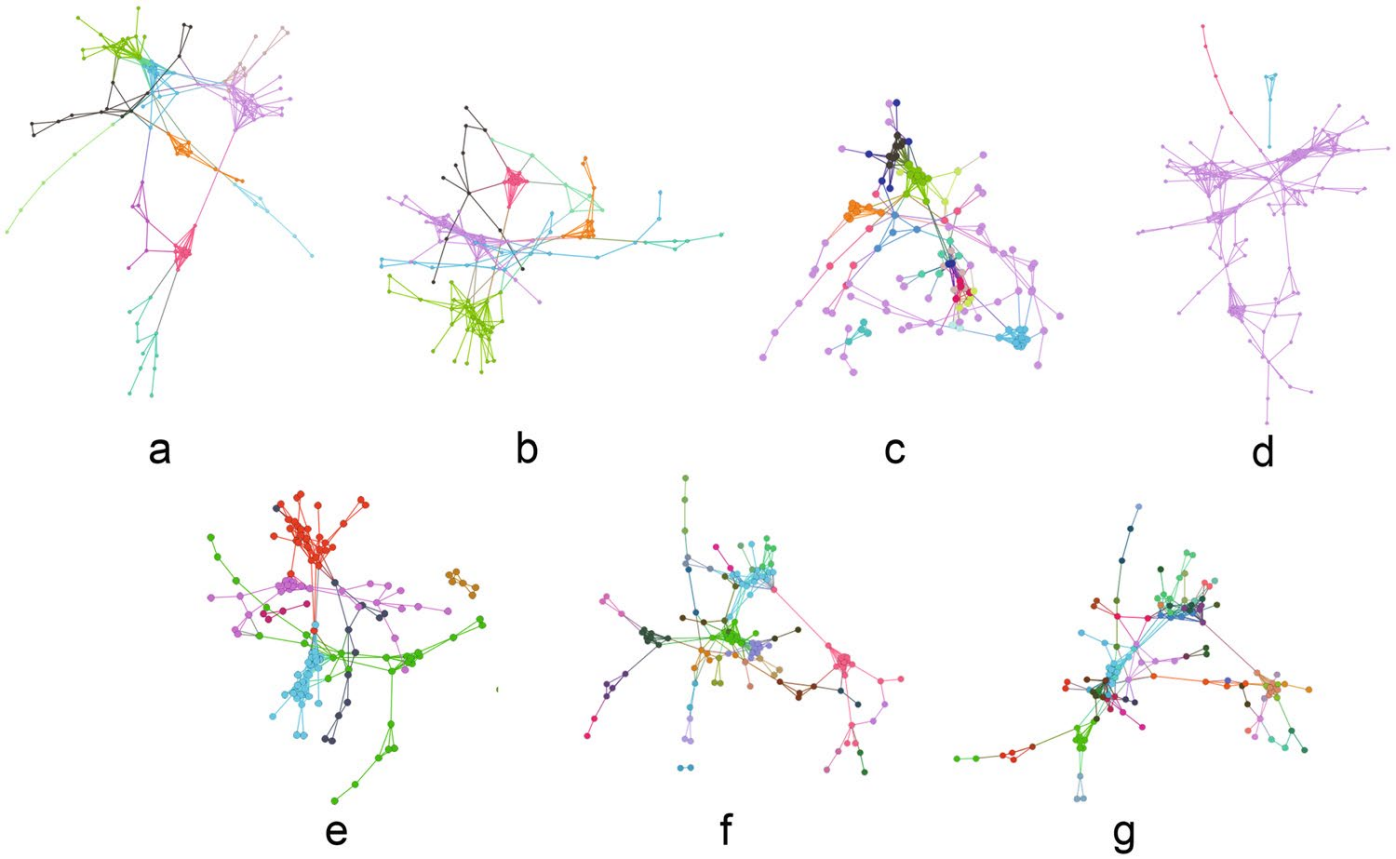
Results of seven community-detection algorithms on Twitter Event Networks. (**a**) Modularity Function in multiplex networks; (**b**) BGLL for multiplex networks; (**c**) Tensor Decomposition for multiplex networks; (**d**) Bridge Detection; (**e**) Modularity-driven Ensemble-Based Community Detection; (**f**) Multidimensional Label Propagation Algorithm; (**g**) Multilayer Local Community Detection.

We analysed the relationships among events detected from Twitter. The tweet stream is captured through the Tweet API^68^. Tweets are clustered using similar keywords to detect the Twitter events. Each node in the network represents a Twitter event. We build the three networks below to construct the multiplex relationship among Twitter events (see Supplementary Note 4). The results of the four community-detection algorithms are visualized in Fig. 4. To facilitate the visualization, we combined the three networks into one network. Nodes of the same colour represent a community, meaning that these nodes correspond to the same event. In Fig. 4, BD results are not obviously better than those of the six other algorithms, but the other six algorithms could not be judged intuitively. Therefore, we present the community quality measures in Table 2. As listed in Table 2, the three measures for our method is much higher than those of the other methods, especially redundancy, which is 0.16, and the ground truth, which is 0.2. The high redundancy and node similarity lead to the high accuracy (72%) of our method, which is considerably higher than the accuracies achieved by BD and TD. BGLLMN also attains high accuracy (72%) because it is based on the novel modularity of a single network. When we combine these three networks into one network, some connection information is lost, but these losses are determined by the network structures. When the losses are relatively low, BGLLMN can exhibit good performance; however, the community redundancy of BGLLMN (0.07) is still much lower than that of our method (0.16). Also, the three new algorithm (M-EMCD, MDLPA and ML-LCD) perform the relative high accuracy but low redundancy, which means that our modularity for multiplex network catch the redundancy of the network.

**Table 2:** Community Quality Measures of Twitter Event Networks.

Community Detection Algorithms	Node Similarity	Community Redundancy	Accuracy
Modularity Function in multiplex networks	0.30	0.16	72%
BGLL for multiplex networks	0.26	0.07	72%
Bridge Detection	0.15	0.07	55%
Tensor Decomposition	0.22	0.11	34%
M-EMCD	0.20	0.07	71%
MDLPA	0.29	0.07	70%
ML-LCD	0.29	0.06	69%
Groundtruth	0.42	0.21	100%

### Noordin Terrorist Relationship Networks

**Figure 5:**
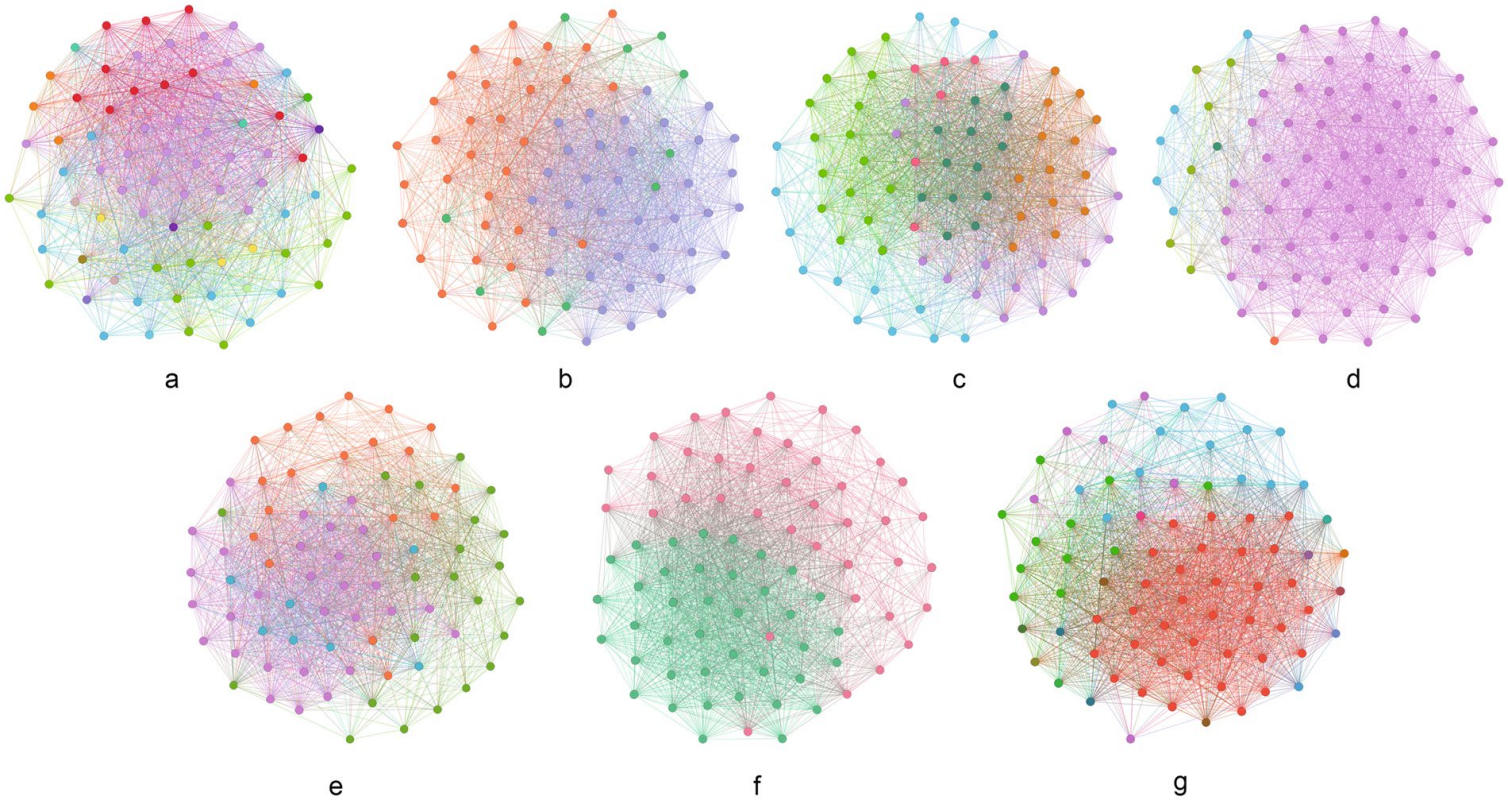
Results of seven community-detection algorithms on Noordin Terrorist Relationship Networks. (**a**) Modularity Function in multiplex networks; (**b**) BGLL for multiplex networks; (**c**) Tensor Decomposition for multiplex networks; (**d**) Bridge Detection; (**e**) Modularity-driven Ensemble-Based Community Detection; (**f**) Multidimensional Label Propagation Algorithm; (**g**) Multilayer Local Community Detection.

Using the Noordin terrorist network data^69,70^, we constructed the multiplex terrorist relationship networks based on six relationships between terrorists. Each node in the network represents a terrorist (see Supplementary Note 4). The results of the four community-detection algorithms are visualized in Fig. 5. To facilitate visualization, we combined the six networks into one network. Nodes of the same colour represent a community, meaning that these nodes likely belong to the same terrorist organization. In Fig. 5, shows almost the same conclusion as in the previous test—that the results of BD are not better than those of the three other methods. The number of communities found by BGLLMN is less than that for TD and for our method. This result may cause high node similarity (shown in Table 3) because most of the nodes are divided into the same community, which results in pairs of nodes having more common neighbours (see Supplementary Note 3). However, according to Table 3, our method still has the highest community redundancy (0.30) and accuracy (27%), which again shows that the communities in real multiplex networks always have high redundancy. Three new algorithms still have a good performance on node similarity and accuracy but low redundancy. In addition, the accuracy of all the algorithms is low because there are many noise data and the ground truth may not agree with the network structure. Therefore, we could judge only whether the algorithm is good or bad through comparisons. Based on the results, our method performs better than do the others (see Table 3).

**Table 3:** Community Quality Measures of Terrorist Relationship Networks.

Community Detection Algorithms	Node Similarity	Community Redundancy	Accuracy
Modularity Function in multiplex networks	0.71	0.30	63%
BGLL for multiplex networks	0.75	0.19	50%
Bridge Detection	0.63	0.11	48%
Tensor Decomposition	0.69	0.25	50%
M-EMCD	0.67	0.22	56%
MDLPA	0.68	0.18	49%
ML-LCD	0.71	0.26	57%
Groundtruth	0.55	0.26	100%

### Students’ Cooperation Social Networks

**Figure 6:**
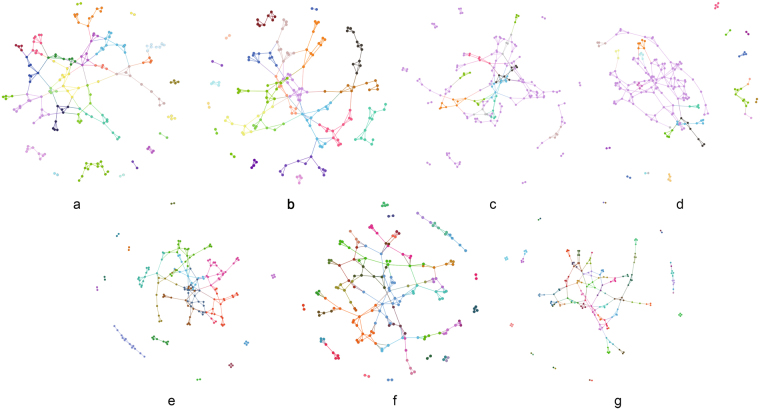
Results of seven community-detection algorithms on Students’ Cooperation Social Networks. (**a**) Modularity Function in multiplex networks; (**b**) BGLL for multiplex networks; (**c**) Tensor Decomposition for multiplex networks; (**d**) Bridge Detection; (**e**) Modularity-driven Ensemble-Based Community Detection; (**f**) Multidimensional Label Propagation Algorithm; (**g**) Multilayer Local Community Detection.

The Students’ Cooperation Social Networks dataset is constructed based on a Computer and Network Security course given at Ben-Gurion University of the Negev^71^ in which students are required to submit a paper to specific web sites. We built the students’ cooperation social networks based on the course website log. Each node in the network represents a student (see Supplementary Note S4). The results of the four community-detection algorithms are visualized in Fig. 6. To facilitate the visualization, we combined the six networks into one network. Nodes of the same colour represent a community, meaning that these nodes likely belong to the same group. In the Students’ Cooperation Social Networks, the first network represents the partner relationships between pairs of students (see Supplementary Note S4). We use these disconnected communities as the ground truth and the other two networks as noise data. Intuitively, BGLLMN and our method perform better than do BD and TD, as shown in Fig. 6, because the community discrimination in the BD and TD results is insufficient. From the measure comparison in Table 4, we can see directly that BD and TD have lower values on all three measures than do the other five methods. Though the MDLPA has the highest redundancy (0.21), the accuracy of it (45%) is much lower than our method (57%). This is because the MDLPA detect 29 communities, which is less than our method (49). Moreover, there are 51 communities in the real network. Our method has the highest values of node similarity (0.34) and accuracy (59%). The results show that in an environment with noisy networks, our method demonstrates a strong anti-noise capability.

**Table 4:** Community Quality Measures of Students’ Cooperation Social Networks.

Community Detection Algorithms	Node Similarity	Community Redundancy	Accuracy
Modularity Function in multiplex networks	0.34	0.14	59%
BGLL for multiplex networks	0.28	0.14	48%
Bridge Detection	0.22	0.09	41%
Tensor Decomposition	0.26	0.11	45%
M-EMCD	0.21	0.13	41%
MDLPA	0.34	0.21	45%
ML-LCD	0.33	0.13	57%
Groundtruth	0.57	0.13	100%

### Global Terrorism Networks

**Figure 7:**
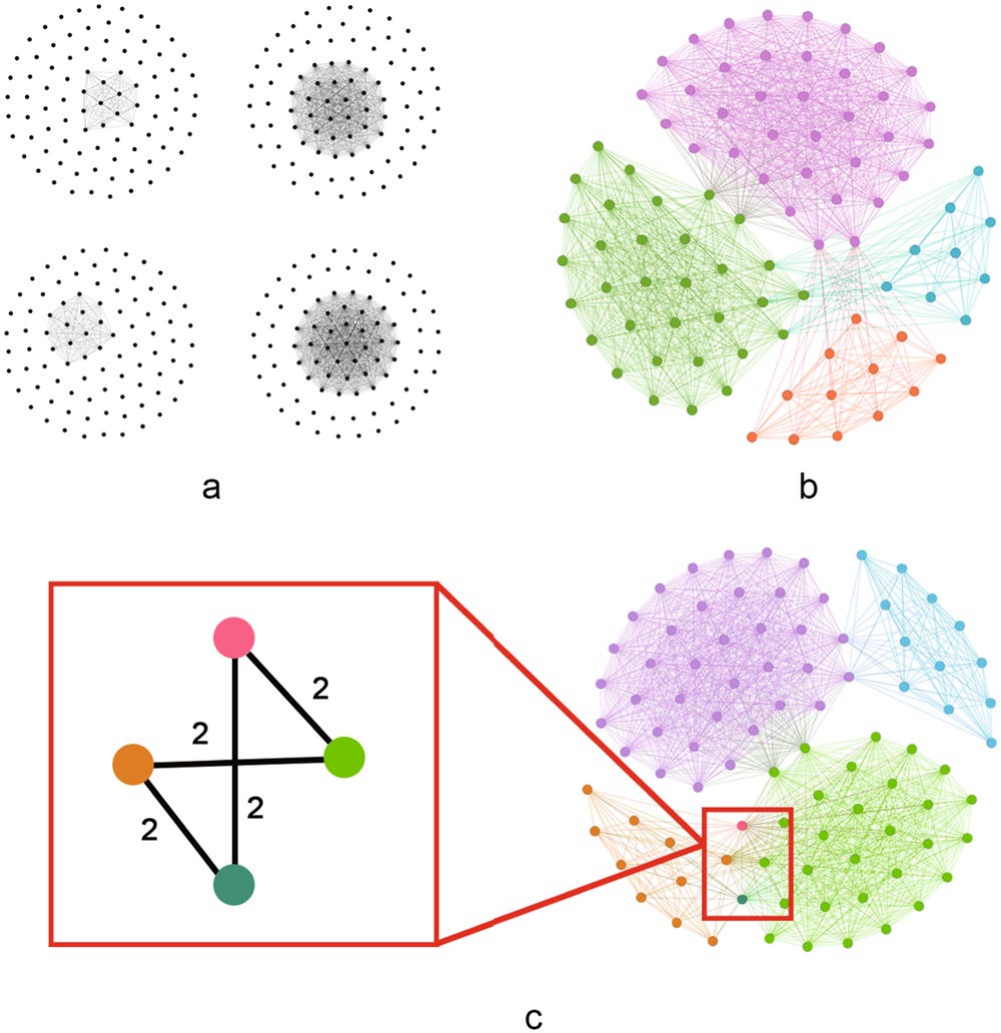
Experimental results on Global Terrorism Dataset networks. (**a**) Four networks in which a terrorist organization is connected to another one if they have performed an attack in the same country. (**b**) The same results of community detection via six algorithms (BGLLMN, BD, TD, M-EMCD, MDLPA, ML-LCD) on the projection networks in (a). (**c**) The results of community detection via MFMN.

From the database of global terrorism^72^, we created four networks in which one terrorist organization is connected to another if they both performed an attack in the same country during the same year. Each node in the network represents a terrorist organization (see Supplementary Note 4). Nodes of the same colour represent a community, meaning that these nodes performed an attack in the same country. In Fig. 7a, there are four complete sub-graphs in each network. The other single nodes in the networks are organizations that did not attack during this year and in this country; therefore, there is no connection between them. We find that the six other community-detection algorithms (BGLLMN, BD, TD, M-EMCD, MDLPA, ML-LCD) obtained the same results: the community is divided into these four networks, as shown in Fig. 7b. When we combine these four networks into one network, none of the edges are redundant except for the edges in the red box in Fig. 7c, which displays the results of our algorithm. The nodes in the red box are connected to each other by edges with a weight of 2. The four nodes are divided into different communities, meaning that our algorithm could reveal the organizations that performed attacks twice in two countries. More generally, our community detection function captures the edges with high redundancy, leading to the high redundancy of communities. This is because we achieved high accuracy on the three multiplex networks described above (see Tables 2, 3, and 4).

## Discussion

The results reported in the preceding section demonstrate the advantageous community detection performance on real-world multiplex networks based on the NMR. In all three networks, our algorithm obtained considerably higher values on all three measures: node similarity, community redundancy and accuracy with ground truth. In turn, the meaningful community structures with different redundant parts of multiplex networks are revealed by our NMR, as demonstrated on the fourth multiplex network. Therefore, we have shown that our framework accurately reflects the community quality and that it maximally preserves the community redundancy, which indicates that it could be a reasonable function for community detection in multiplex networks.

The general conclusion from the results presented in this paper is that communities in real-world networks always have much higher redundancy, which verifies the importance of capturing the NRD in multiplex networks. Both the theoretical and experimental results show that NRD is a reasonable measure for describing the connection relationships of multiplex networks. With regard to a single network, NRD automatically degenerates to the node degree. Therefore, NRD is a more general and fundamental measure that includes the node degree as a specific case for single networks. Indeed, this measure can be used in systems with arbitrary nodes, edges and layers—not only in social networks as described above but also in other multi-layer networks such as traffic networks, metabolic networks, epidemic networks and Internet topology.

In a more general sense, the NMR is a general null model for any multiple-relationship system such as the social networks utilized above. We developed the NMR and its higher-order representation using the basic configuration method based on the NRD. The rationality of the NMR can also be explained by the traditional random-walk theory. The connection between the 0-order NMR and the original networks is almost completely random, except for size. As the order increases, the model gradually becomes closer to the original multiplex network, and as more attributes match those of the original network, the model becomes the same as the original multiplex network. For different purposes, the order of the NMR can be controlled to guarantee the connection similarity to the original network, and other properties of the original network can be exposed by the comparison.

The general significance of the NMR is that in addition to community structure, many other specific properties can be revealed through the different orders of the model. These properties, including motif identities, propagation-rate threshold, redundancy-distribution correlations and synchronization-state stability, have already been shown to be important in network science. Additionally, the NMR can be used in directed networks based on in-and-out NRD. For example, a comparison of the number of structures appearing in the NMR with the same in-and-out NRD distribution may help researchers determine whether this higher-order structure is the most important motif in the original multiplex network. Our future work is based on such extensions of our NMR and its high-order representations, which may lead to some problems involving the applications of all systems with multi-relationships that can be described by multiplex networks.

Finally, our null model of multiplex networks provides a powerful tool for the structure analysis of complex systems with multiple relationships. Through comparisons, the specific nature of these multi-relationship systems can be exposed quantitatively by the NMR. We believe that the NMR can give rise to much stronger and more general applications in many areas, including social science, Internet topology, bioscience, engineering, economics, and education, where multi-relationship systems can be described by multiplex networks. To accomplish this, much more work needs to be done to gain a deeper understanding of the model and its high-order representations, such as a determination of the NRD distribution law. We hope that many more attributes of multi-relationship systems can be modelled and analysed through the null model with redundancy for multiplex networks.

## Methods

### Generation of the 1-Order Null Model with Redundancy for Multiplex Network

To generate the one-order NMR, we introduce the random configuration model of multiplex networks based on the configuration model in single networks.

The random configuration model is constructed as follows:

1. Divide the edges into *M* groups according to their ERC values. Because each edge *e*_*ij*_ may have different ERC values between different nodes, we should assign them separately according to the ERC to ensure that the NRD of each node is the same as that of the original one. The number of edges in each group *μ*^*m*^ is defined by the number of edges in the *m*-ERC, where 0 ≤ *m *< *M*.
2. Assign the *M* groups of edges randomly to the models that have the same size as the original multiplex network. There are *M* layers of the network and *m* + 1 edges in the (*m* + 1)-th groups in which the *m*-ERC equals *m* (0 ≤ *m *< *M*). Therefore, the edge assignment process is an (*m* + 1)-combination problem. The total number of assignments in (*m* + 1) groups is ${C}_{M}^{m+1}=\frac{M!}{[M-(m+1)]!\times (m+1)!}$, which can also be written as $$(\begin{array}{c}M\\ m+1\end{array})$$. The number of assignments in a specific layer of network *A*_*i*_ is ${C}_{m+1}^{1}$. Therefore, the probability that network *A*_*i*_ is assigned an *m*-ERC edge is ${p}_{m}({A}_{i})=\frac{{C}_{m+1}^{1}}{{C}_{M}^{m+1}}$.
3. Assign the *m*-ERC edges to nodes *i* and *j*. For node *i*, there are $(m+1){r}_{i}^{m}$ edges that can be assigned for the *m*-ERC edges. For the entire network, a total of 2 *μ*^*m*^ edges can be assigned for the *m*-ERC edges. We consider that the process of one edge selecting the two end nodes is independent. Therefore, the probability that node *i* and node *j* in network *A*_*i*_ are assigned an *m*-ERC edge is ${p}_{{A}_{i}}(i,j)=\frac{(m+1){r}_{i}^{m}}{2{\mu }^{m}}\times \frac{(m+1){r}_{j}^{m}}{2{\mu }^{m}}$.
4. Assign all the edges to the model. Note that the processes of edge assignment to networks and to nodes are independent. Thus, at the end of the assignment processes, the probability of an edge existing between a node *i* and node *j* in one network is

8 $$p(i,j)^{\prime} =\sum _{m}{p}_{m}({A}_{i})\times {p}_{{A}_{i}}(i,j)=\sum _{m}\frac{{C}_{m+1}^{1}}{{C}_{M}^{m+1}}\frac{{(m+1)}^{2}{r}_{i}^{m}{r}_{j}^{m}}{{(2{\mu }^{m})}^{2}},0\le m < M$$

Thus, in *M* networks, the probability of an edge existing between node *i* and node *j* is:

9 $$p(i,j)=M\sum _{m}\frac{{C}_{m+1}^{1}}{{C}_{M}^{m+1}}\frac{{(m+1)}^{2}{r}_{i}^{m}{r}_{j}^{m}}{{(2{\mu }^{m})}^{2}},0\le m < M$$

### Explanation of Random Travel

We can also generate our NMR based on a random walk under Laplacian dynamics^60^. Here, we suppose there is a traveller who travels randomly from any one node to any other node in a multiplex network, even if the two nodes are in different networks. In contrast to a random walk on a single network, the traveller can travel between different networks only when two nodes are connected in any network. Thus, we call the agent a “traveller” rather than a “walker”.

Because the edges can be divided into *M* groups according to their ERC values, we can divide the multiplex network into *M* layers in which the ERC is the same for all edges in each layer. Thus, the traveller can travel among all the layers of the multiplex network, which means that the traveller can choose edges with different ERC values to travel between layers. In the *m*-layer, where the ERC values of all the edges are equal to *m*, the probability of the traveller travelling from node *i* to node *j* in the model is

10 $$p{(j|i)}_{m}=\frac{{C}_{m+1}^{1}}{{C}_{M}^{m+1}}\frac{(m+1){r}_{j}^{m}}{2{\mu }^{m}}$$

The random travel process, similar to the random walk process, is a Markov process. When the process is stable in each layer, the steady-state probability distribution is

11 $${p}_{i}^{m\ast }=\frac{(m+1){r}_{i}^{m}}{2{\mu }^{m}}$$

Thus, the joint probability of traveler traveling from node *i* to *j* in one network in the model is:

12 $$p(i,j)^{\prime} =\sum _{m}p{(j|i)}_{m}\times {p}_{i}^{m\ast }=\sum _{m}\frac{{C}_{m+1}^{1}}{{C}_{M}^{m+1}}\frac{{(m+1)}^{2}{r}_{i}^{m}{r}_{j}^{m}}{{(2{\mu }^{m})}^{2}},0\le m < M$$

In *M* networks, the probability of an edge existing between node *i* and node *j* is

13 $$p(i,j)=M\sum _{m}\frac{{C}_{m+1}^{1}}{{C}_{M}^{m+1}}\frac{{(m+1)}^{2}{r}_{i}^{m}{r}_{j}^{m}}{{(2{\mu }^{m})}^{2}},0\le m < M$$

The probability of each edge occurring in the random travel model is the same as that in the random configuration model. Thus, the two models are unified for multiplex networks, which verifies the correctness and validity of our NMR.

### Fast Algorithm of Community Detection based on the Multiplex Networks Modularity Function

In the era of big data, the scale of networks is becoming increasingly large. Thus, we propose a new fast algorithm for community detection based on the multiplex networks modularity function in large networks (FCDMNN). This work is based on the work of V. D. Blondel^61^. The steps in the algorithm are as follows:

1. Initialization: We regard each node in the multiplex network as a community. Thus, the number of communities is *N*, which also denotes the number of nodes.
2. Traverse each node *i* in the multiplex network to find all the nodes connected with node *i*. Compute the modularity increment Δ*Q* of each neighbouring node *k* of node *i*. Δ*Q* is defined as follows:
  14 $${\rm{\Delta }}{Q}_{ij}=\frac{1}{2\mu }\{\sum _{k\in {g}_{j}}[{w}_{ik}-P(i,k)]-\sum _{k\in {g}_{i}}[{w}_{ik}-P(i,k)]\}$$
  where $P(i,k)=2\mu M\sum _{m}\frac{{C}_{m+1}^{1}}{{C}_{M}^{m+1}}\frac{{(m+1)}^{2}{r}_{i}^{m}{r}_{k}^{m}}{{(2{\mu }^{m})}^{2}}$ is the expected number of edges of 1-order NMR.
3. Find the community *C*_*k*_of node *k* with the maximum Δ*Q*. Add node *i* to community *C*_*k*_.
4. Repeat steps (2) and (3) until the communities no longer change.
5. When step (4) is complete, regard each community as a node. The edges within each community can be regarded as the loopback weighted edges of the new node. Here, the weight is the number of edges within the community to which the node belongs.
6. The edges between two communities can be regarded as the weighted edges of the two new nodes. Here, the weight is the total number of edges between the two communities to which the nodes belong.
7. Repeat steps (2)–(5) until the communities no longer change.

The time complexity of FCDMNN is $O(N\times \sum _{m}{r}_{max}^{m})$, where ${r}_{max}^{m}$ denotes the maximum *m*-order NRD and *N* refers to the number of nodes. Compared with the BGLL algorithm, the time complexity of our algorithm is slightly higher. However, for large networks, $\sum _{m}{r}_{max}^{m}$ is far less than the number of nodes *N*. Thus, the time complexities of the two algorithms are both *O*(n). However, our algorithm is acceptable for multiplex networks and the quality of the resulting communities is better compared to other multiplex-network community detection algorithms.

## Electronic supplementary material

Supplementary Information
