# Supplementary material for: Null Model and Community Structure in Multiplex Networks
Source: Sci Rep. 2018 Feb 19;8:3245. doi: 10.1038/s41598-018-21286-0 (PMC5818485; doi:10.1038/s41598-018-21286-0)
Supplement: Supplementary file 1 — Supplementary Information [file 41598_2018_21286_MOESM1_ESM.pdf]

Supplementary Information for Null Model and Community Structure in Multiplex Networks

Xuemeng Zhai[1], Wanlei Zhou[2], Gaolei Fei[1], Weiyi Liu[1], Zhoujun Xu[3], Chengbo Jiao[3], Cai Lu[1], Guangmin Hu[1,4]*

[1] School of Communication and Information Engineering, University of Electronic Science and Technology of China, Chengdu, China.

[2] Faculty of Science, Engineering and Built Environment, Deakin University, 221 Burwood Highway, Burwood, VIC 3125, Australia

[3] Beijing Information Technology Institute, Beijing, China

[4] Center for Information Geoscience, University of Electronic Science and Technology of China, Chengdu, China

* Correspondence and requests for materials should be addressed to Guangmin Hu (email: hgm@uestc.edu.cn).

This PDF file includes:

Supplementary Note 1. Null Model and Its High-Order Expression
Supplementary Note 2. Other Algorithms for Multiplex Networks Communities Detection
Supplementary Note 3. Measures for Community Quantitative Comparison
Supplementary Note 4. Networks
Supplementary Note 5. Modularity in Single Network
Supplementary Figure 1. Twitter Event Networks.
Supplementary Figure 2. Terrorist Relationship Networks.
Supplementary Figure 3. Students' Cooperation Social Networks.
Supplementary Table 1. The summary of dK-series

Supplementary Note 1. Null Model and its Higher-Order Expression

In network science, the null model is a stochastic network that matches the original networks in terms of some of its structural features, but is otherwise taken to be an instance of a random network. The null model is used as a term of comparison, to verify whether the network in question displays some feature such as community structure. One null model proposed by Newman[1] consists of a randomized version of the original network, where edges are rewired at random, under the constraint that the expected degree of each vertex must match the degree of the vertex in the original network.

In Priya Mahadevan's research[2], the null model of the network is divided into dK-series to describe and constrain random network *s* in successively finer detail. They denote by dK-networks the sets of networks constrained by given values of dK-distributions. Producing a family of 0K-networks for a given input network requires reproducing only the average node degree of the original network, while producing a family of 1K-networks requires reproducing the original network's node-degree distribution, namely, the 1K-distribution. 2K-networks reproduce the joint degree distribution, namely, the 2K-distribution, of the original network, which describes the probability that two nodes of degrees k and k′ are connected. 3K-networks consider interconnectivity among triples of nodes, and so forth. Generally, the set of (d + 1) K-networks is a subset of dK-networks. The summary of the dK-series null model is shown in Table S1.

Actually, the dK-series null model is a network that matches the original networks in terms of size and d-order degree distribution but is otherwise taken to be an instance of a random network.

Supplementary Note 2. Other Algorithms for Multiplex Networks Communities Detection

To evaluate the performance of our method, we compare the modularity of multiplex networks to those of existing, popular community-detection methods. We chose three representative algorithms: BGLL for multiplex networks (BGLLMN)[5,6], which is shown to outperform all other known community-detection methods in terms of computation time; Bridge Detection (BD)[7], which can be used to detect how strongly the neighborhoods of two nodes linked by an edge are connected; Tensor Decomposition (TD)[8], which subjects multiplex networks to single-relational network analysis; Modularity-driven Ensemble-Based Community Detection (M-EMCD)[9], which uses the modularity for multi-slice network to drive the classic ensemble-based community detection method; Multidimensional Label Propagation Algorithm (MDLPA)[10], which could give the communities and their most relevant layers; Multilayer Local Community Detection (ML-LCD)[11], which exploits both internal and external connectivity

of the nodes in the community being constructed for a given seed.

BGLL for multiplex networks: The basic method for community detection in multiplex networks is to project the multiplex into a weighted graph. For example, let A^i be the adjacency matrix of network i in a multiplex network. The adjacency matrix of the weighted projection of the multiplex network is given by $W = \frac{1}{M}\sum_{m=1}^{M} A^i$, which is called the "Projection Average" of the multiplex network. BGLL[5, 6] is a simple method to extract the community structure of large networks. The algorithm is a heuristic method that is based on modularity optimization, which is shown to outperform all other known community-detection methods in terms of computation time. The quality of the detected communities is high, as measured by the modularity. The limitation of the method is the storage of the network in main memory, rather than the computation time. BGLL reveals a complete hierarchical community structure for the network, with each level of the hierarchy being given by the intermediate partitions found at each pass. Here, we use the BGLL in the weighted projection of the multiplex networks to detect the communities.

Bridge Detection: Bridge detection is a method based on a measure called multi-layered edge clustering coefficient (CLECC)[7]. The CLECC measure is slightly modified and expresses the similar neighbors' interconnectivity for multi-layered social networks or multiplex networks. This measure can be used to determine how strongly the neighborhoods of two nodes linked by an edge are connected. Consequently, this measure can be utilized to detect communities in the multiplex social network. A bridge in a network refers to an edge with low CLECC, like the low-traffic links between two services, where the absence of these links separates the nodes into isolated communities. These communities can be detected by projecting the multiplex network onto a weighted network with the CLECC and determining the bridges from the projection. Alternatively, one can remove them by defining a multiplex bridge to obtain the desired partitions.

Tensor Decomposition: The multiplex-network path algebra presented in this article operates on an n × n × m tensor representation of a multiplex network[8]. By means of a series of operations on two-way "slices" of this tensor, a "semantically rich" n × n single-relational path matrix can be derived. The resulting path matrix represents a single-relational network. This single-relational network may then be subjected to any of the known single-relational network analysis algorithms. Thus, the presented path algebra can be used to expose multiplex networks to single-relational network analysis algorithms.

Modularity-driven Ensemble-Based Community Detection: The method is proposed by Andrea Tagarelli[9]. It finds consensus community structures that not only capture prototypical community memberships of nodes, but also preserve

the multilayer topology information and optimize the edge connectivity in the consensus via modularity analysis. The approach has three steps: first, it detect the communities in each layer separately using the algorithm of the single network; second, aggregate communities in each layer to build the co-association construction; finally, optimize the community connectivity based the modularity. The consensus function is learned via modularity-based optimization instead of being simply based on the sharing of a certain minimum percentage of clusters in the ensemble.

Multidimensional Label Propagation Algorithm: The approach is proposed by Boutemine. It allows automated identification of communities and their sub-dimensional spaces using a novel objective function and a constrained label propagation-based optimization strategy. The MLPA aims to maximize the number of relevant within-community links while keeping track of the most relevant dimensions by leveraging the relevance of dimensions at the node level. The relevant dimensions of communities are identified based on the neighbors of members in the communities. The approach is capable of identifying meaningful communities that reflect hidden functional and organizational characteristics of real world systems.

Multilayer Local Community Detection: The approach is proposed by Interdonato and is a method for local community detection in multilayer networks. The method exploits both internal and external connectivity of the nodes in the community being constructed for a given seed, while accounting for different layer-specific topological information. To obtain the whole result of community detection, we should traverse all the nodes in the graph and get the every local community. It employs a greedy heuristic that considers both internal and external connectivity, following an unsupervised paradigm that exploits layer-specific topological information.

Supplementary Note 3. Measures for Community Quantitative Comparison

There are some subtle aspects to consider when comparing disparate community algorithms. Some methods find excellent communities (high quality) but low redundancy. Others find medium-quality communities but classify the majority of the network. Since it is difficult and unfair to compare all methods along any one of these directions, we have introduced three measures to fairly account for these differences while also allowing a researcher to focus on the individual aspects.

We study three distinct aspects of the quality of the communities found—the quality measures are based on ground truth and the theory of community quality.

Node Similarity: As we all know that two nodes with the same neighbors are more likely to be divided into a community, node similarity refers to the

common neighbors of two nodes in the same community. We build the node similarity as a parameter to measure the quality of community detection results because it reflects the similarity of two nodes in the same community. After normalization, we define node similarity as:

$$Sim = \frac{1}{N}\sum_{i,j}\frac{|n(i)\cap n(j)|}{|n(i)\cup n(j)|}\delta(g_i, g_j) \qquad \text{(S1)}$$

where N is the node number, $n(i)$ refers the neighbor number of node i, $\delta(g_i, g_j) = 1$when i and j are in the same community and 0 otherwise. This parameter is very similar to the one used in link community to cut the edges[12].

Community Redundancy: The measure is proposed by Michele Berlingerio[3] to capture the phenomenon for which a set of nodes that constitute a community in a network tend to constitute a community also in other networks. As a parameter to measure the results, community redundancy could reflect the best result where the nodes in the same community are more likely to connect in all networks. The community redundancy ρ_C is defined as:

$$\rho_C = \frac{1}{N_C}\sum_{(c\in C)}\frac{\sum_{i,j\in\overline{P_c}} rm_{ij}}{M\times|P_c|} \qquad \text{(S2)}$$

where N_C is the communities number, P_c is set of two nodes connected by at least in one network in community c, P_c is set of two nodes connected by at least in two networks in community c, rm_{ij} is the redundancy of edges between node i and j.

Accuracy: The measure is built based on the ground truth. It is the main parameter to measure the quality of communities. It means the accuracy of the experimental results compared with the real community. We define the measure as the percentage of nodes which are divided into the correct community. For example, if there are 8 nodes in community c in experimental results and only 5 nodes in the same community in real, the accuracy of these nodes is 5/8. With normalization, the accuracy is defined as:

$$a = \frac{N_{right}}{N} \times 100\% \qquad \text{(S3)}$$

where N_{right} is the number of nodes that are divided into right communities, and N is the node number.

Supplementary Note 4. Networks

Twitter Event Networks (Fig. 1):

- Node: Events detected from Twitter

Network 1. Key Words Similarity Network:

- Edge: The similarity of key words of two events
- Edge Forming Conditions: If the cosine similarity between two events' words vector is more than 0.3, we add an edge to the two nodes.

Network 2. Location Relationship Network:

- Edge: The similarity of location of two events
- Edge Forming Conditions: If two events occurred in the same place, we add an edge to the two nodes.

Network 3. Tweets Correlation Network:

- Edge: The correlation of tweets set of two events
- Edge Forming Conditions: If the same tweets between two events are more than 10, we add an edge to the two nodes.

Noordin Terrorist Relationship Networks (Fig. 2):

- Node: Terrorists

Network 1. Organization Relationship Network:

- Edge: The organization relationship of two terrorists
- Edge Forming Conditions: If the two terrorists belong to the same organization, we add an edge to the two nodes.

Network 2. Education Background Network:

- Edge: The education relationship of two terrorists
- Edge Forming Conditions: If the two terrorists have the same education background (study in the same university), we add an edge to the two nodes.

Network 3. Communication Relationship Network:

- Edge: The communication relationship of two terrorists
- Edge Forming Conditions: If the two terrorists communicate with each other frequently, we add an edge to the two nodes.

Network 4. Action Network:

- Edge: The action relation of two terrorists
- Edge Forming Conditions: If the two terrorists are involved in the same terrorist attack, we add an edge to the two nodes.

Network 5. Friendship Network:

- Edge: The friend relationship of two terrorists
- Edge Forming Conditions: If the two terrorists are friends, we add an edge to the two nodes.

Network 6. Logistics Relationship Network:

- Edge: The logistics relationship of two terrorists
- Edge Forming Conditions: If the two terrorists have same logistics relationship, we add an edge to the two nodes.

Students' Cooperation Social Networks (Fig. 3):

- Node: Students

Network 1. Partners Relationship Network (Explicit Relation):

- Edge: The partner relationships of two students
- Edge Forming Conditions: If the two students are partners marked in the paper, we add an edge to the two nodes.

Network 2. Computer Sharing Network:

- Edge: The computer sharing relationships of two students
- Edge Forming Conditions: If the two students share a computer to submit the paper, we add an edge to the two nodes.

Network 3. Time sharing Network:

- Edge: The temporal correlation of submitting the paper by two students
- Edge Forming Conditions: If the twos students turn in report in the same time, we add an edge to the two nodes.

Global Terrorism Networks (Fig. 7 in main text):

- Node: Terrorism Organizations

- Edge: Co-attack from the same country
- Edge Forming Conditions: if two terrorist organization have performed an attack in the same country, in the same year, we add an edge to the two nodes.

Supplementary Note 5. Modularity in a Single Network

In 2006, Newman proposed a function called modularity to quantify communities in a single network[4]. The modularity refers to the difference between the number of edges in communities of real network and expected number in the network model. The modularity Q is defined as

Q = (number of edges within communities) − (expected number of such edges) (S4)

The first-order null model, which is similar to the configuration model in a large network, is used to calculate the modularity in Newman's study. Given the network adjacency matrix A, where the element a_{ij} describes a direct connection between nodes i and j, one can construct modularity as

$$Q = \frac{1}{2m}\sum_{ij}[a_{ij} - P_{ij}]\delta(g_i, g_j) = \frac{1}{2m}\sum_{ij}\left[a_{ij} - \frac{k_i k_j}{2m}\right]\delta\left(g_i, g_j\right) \quad \text{(S5)}$$

where P_{ij} is the expected number of edges between node i and node j in the null model; $\delta\left(g_i, g_j\right) = 1$ if node i and node j are divided into the same community and 0 otherwise; and k_i is the degree of node i and refers to the number of edges in the network. This modularity leads to a number of possible algorithms for detecting community structure[13-16]. The function creates a precedent for community discovery.

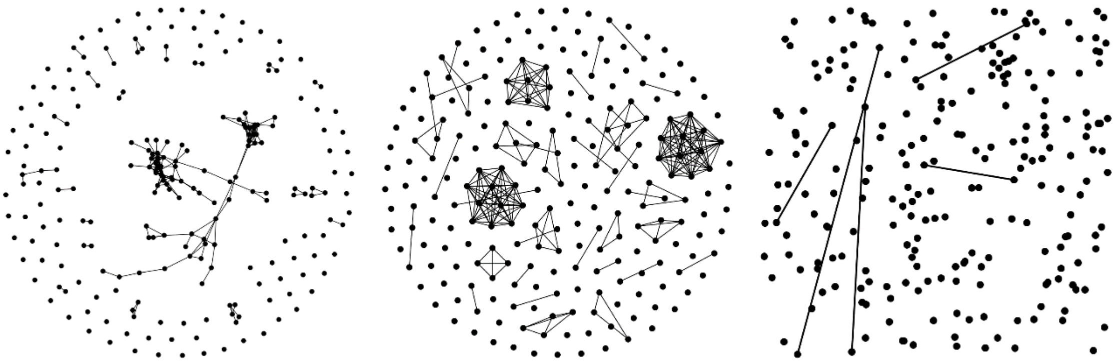

Supplementary Figure 1: Twitter Event Networks. (**a**) Key Words Similarity Network; (**b**) Location Relationship Network; (**c**) Tweets Correlation Network

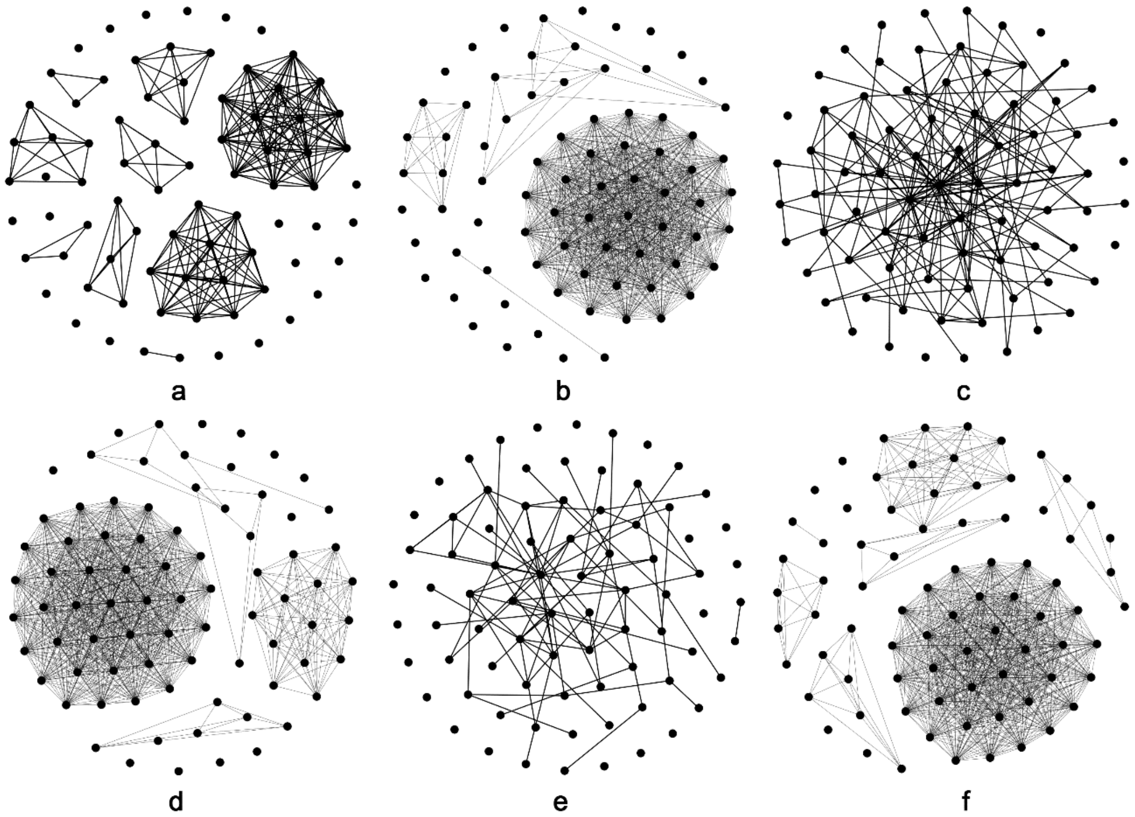

Supplementary Figure 2: Terrorist Relationship Networks. (**a**) Organization Relationship; (**b**) Education Background; (**c**) Communication Relationship; (**d**) Action; (**e**) Friendship; (**f**) Logistics Relationship;

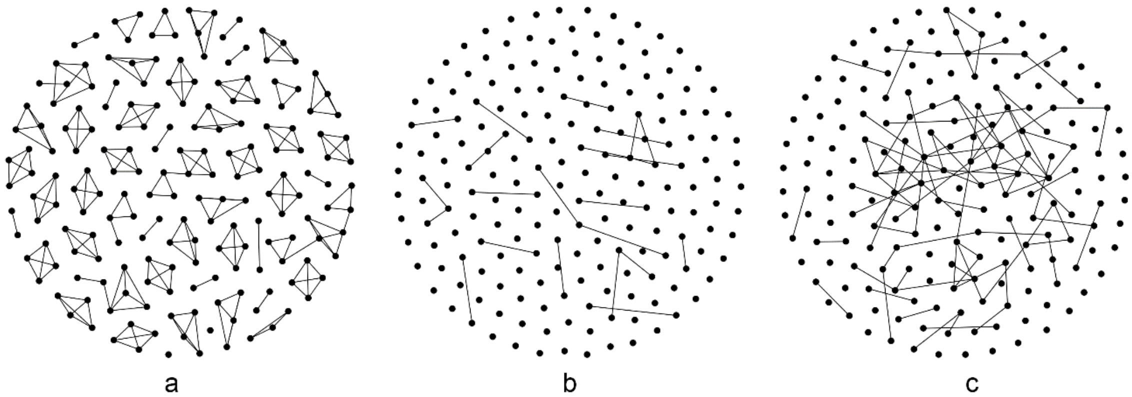

Supplementary Figure 3: Students' Cooperation Social Networks. (**a**) Partners Relationship Network; (**b**) Computer Sharing Network; (**c**) Time sharing Network

Supplementary Table 1: The summary of dK-series.

Supplementary Table 1: The summary of dK-series.		
**Tag *k*-order**	**Property symbol**	***k*-order -distribution**
0K	P_0	$\bar{k}$
1K	*P1*	$P(k)$
2K	*P2*	$P(k_1, k_2)$
3K	*P3*	$P(k_1, k_2, k_3)$
…		
nK	*Pn*	$P(k_1, k_2, \ldots, k_n)$

References and Notes

1. Newman M E, Strogatz S H, Watts D J. Random graphs with arbitrary degree distributions and their applications. *Physical Review E Statistical Nonlinear & Soft Matter Physics*, 64(2 Pt 2):359-382 (2001).
2. Mahadevan P, Krioukov D, Fall K, et al. Systematic topology analysis and generation using degree correlations. *Acm Sigcomm Computer Communication Review*, 36(4):135-146 (2006).
3. Berlingerio M, Coscia M, Giannotti F. Finding and Characterizing Communities in Multidimensional Networks, *International Conference on Advances in Social Networks Analysis and Mining. IEEE Computer Society* :490-494 (2011).
4. Newman M E. Modularity and community structure in networks. *Proceedings of the National Academy of Sciences of the United States of America*, 103(103):8577-8582 (2006).
5. Blondel V D, Guillaume J L, Lambiotte R, et al. Fast unfolding of communities in large networks. *Journal of Statistical Mechanics Theory & Experiment*, 2008(10):155-168 (2008).
6. Loe C W, Jensen H J. Comparison of communities detection algorithms for multiplex. *Physica A Statistical Mechanics & Its Applications*, 431:29-45 (2015).
7. Bródka, Piotr, Tomasz Filipowski, and Przemysław Kazienko. An introduction to community detection in multi-layered social network. *World Summit on Knowledge Society. Springer Berlin Heidelberg* (2011).
8. Rodriguez M A, Shinavier J. Exposing multi-relational networks to single-relational network analysis algorithms. *Computer Science*, 4(1):29-41 (2009).
9. Tagarelli A, Amelio A, Gullo F. *Ensemble-based community detection in multilayer networks*. Data Mining & Knowledge Discovery, (3):1-38. (2017)
10. Boutemine O, Bouguessa M. Mining Community Structures in Multidimensional Networks. *Acm Transactions on Knowledge Discovery from Data*, 11(4):1-36 (2017).
11. Interdonato R, Tagarelli A, Ienco D, et al. *Local community detection in multilayer networks*. Data Mining and Knowledge Discovery, 31(5): 1444-1479 (2017).
12. Ahn, Yong-Yeol, James P. Bagrow, and Sune Lehmann. Link communities reveal multiscale complexity in networks. *Nature*, 466.7307 (2010): 761-764.
13. Lai D, Lu H, Nardini C. Enhanced modularity-based community detection by random walk network preprocessing. *Physical Review E Statistical Nonlinear & Soft Matter Physics*, 81(6 Pt 2):1741-1741 (2010).
14. Waltman L, Eck N J V. A smart local moving algorithm for large-scale modularity-based community detection. *The European Physical Journal B*, 86(11):1-14 (2013).

15. Zhang S, Zhao H. Normalized modularity optimization method for community identification with degree adjustment. *Physical Review E Statistical Nonlinear & Soft Matter Physics*, 88(5):471-490 (2013).
16. Miyauchi A, Kawase Y. Z-Score-Based Modularity for Community Detection in Networks. *Plos One,* 11(1) (2015).
